# Modulation of electronic structure via dual moiré patterns in twisted 1*T*-TaSe_2_

**DOI:** 10.1073/pnas.2520703123

**Published:** 2026-03-11

**Authors:** Yonghao Liu, Yuan Zheng, Kun Yang, Wenhao Zhang, Zongxiu Wu, Jingjing Gao, Xuan Luo, Yuping Sun, Jin Zhang, Yi Yin

**Affiliations:** ^a^Zhejiang Key Laboratory of Micro-Nano Quantum Chips and Quantum Control, School of Physics, Zhejiang University, Hangzhou 310027, China; ^b^Laboratory of Theoretical and Computational Nanoscience, National Center for Nanoscience and Technology, Chinese Academy of Sciences, Beijing 100190, China; ^c^Key Laboratory of Materials Physics, Institute of Solid-State Physics, Hefei Institutes of Physical Science, Chinese Academy of Sciences, Hefei 230031, China; ^d^Anhui Provincial Key Laboratory of Low-Energy Quantum Materials and Devices, High Magnetic Field Laboratory, Hefei Institutes of Physical Science, Chinese Academy of Sciences, Hefei 230031, China; ^e^Collaborative Innovation Center of Advanced Microstructures, Nanjing University, Nanjing 210093, China

**Keywords:** charge density wave, Mott insulator, moiré control, scanning tunneling microscope

## Abstract

We investigate a twisted bilayer of 1*T*-TaSe_2_ (twist angle $<4^{{}^{\circ}}$) using scanning tunneling microscopy and spectroscopy, revealing that the coexisting twisted atomic lattice and charge density wave (CDW) superlattice generate a dual moiré structure with distinct electronic modulation effects: The topographic moiré pattern stems from atomic lattice twisting modulating CDW intensity, while the twisted CDW superlattice drives a continuous insulator-to-metal transition, as evidenced by electronic gap evolution from large to metallic states. Density functional theory calculations show this transition arises from twist-induced changes in star of David motif stacking. Using the moiré-period gap map as the interlayer potential V(r), we construct a continuum model via its Fourier components $V_{G}$, finding that $V_{G}$ mediates multiple interlayer scattering processes that produce numerous superposition states manifesting as split flat-band pairs with distinct energy gaps. This work elucidates a CDW-twist-based mechanism for electronic control in 1*T*-TaSe_2_ and provides insights into Mott physics and complex electronic phases in related materials.

## Significance

This study reveals a unique electronic control mechanism in twisted bilayer 1*T*-TaSe_2_, wherein a dual moiré pattern—arising from both the atomic lattice and the charge density wave (CDW) superlattice—governs distinct modulations of the electronic structure. By quantifying the low-energy CDW moiré potential through spectroscopic mapping, the research establishes a framework for understanding flat-band physics in twisted CDW systems. The moiré-tunable electronic states also establish 1*T*-TaSe_2_ as a promising platform for exploring correlation effects and competing phases in twisted CDW systems.

The facile exfoliation and inherent tunability of layered two-dimensional (2D) materials, assembled via van der Waals (vdW) forces, make them exceptional platforms for engineering diverse quantum phases at the atomic scale (1). Even when individual monolayers are weakly correlated systems—such as graphene or transition metal dichalcogenides (TMDs) like WS_2_ and WSe_2_—whose properties are well described by single-electron theory, stacking them into homogeneous or heterogeneous multilayer structures can unlock a wealth of emergent phenomena. A key strategy involves introducing a twist angle or lattice mismatch between two monolayers. This generates moiré patterns whose wavelengths significantly exceed that of the atomic lattice (2–8). These long-period moiré superlattices induce profound electronic structure modifications, including band folding and the formation of extremely flat electronic bands. Such flat bands dramatically suppress electron kinetic energy, thereby amplifying the effects of Coulomb interactions and electronic correlations (9, 10). Consequently, the system transitions into novel strongly correlated phases, including states such as the Mott insulator and unconventional superconductivity (2, 3).

**Fig. 1.**
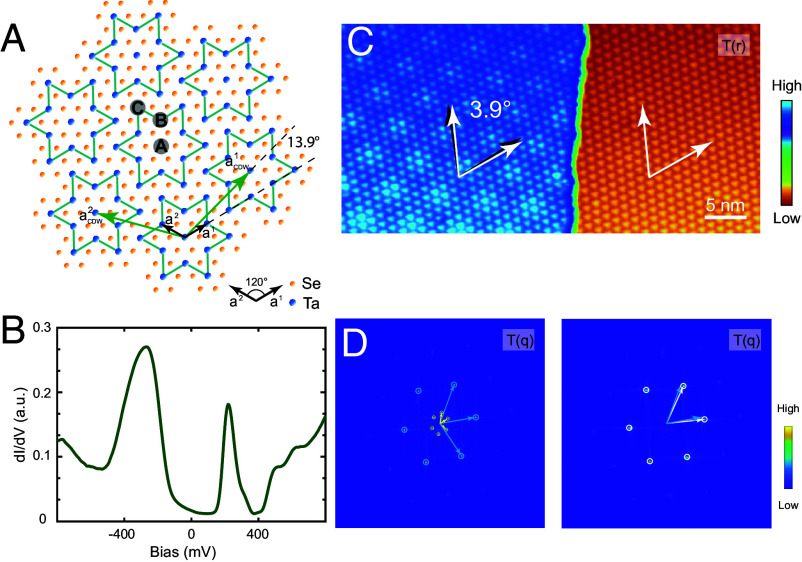
(*A*) *Top* view of the atomic structure of 1*T*-TaSe_2_ with the superimposed SD superlattice. The black and green arrows represent the lattice vectors of the Ta atoms and the SD supercells ($\sqrt{13}\times \sqrt{13}$), respectively. Three Ta atoms are labeled A, B, C, corresponding to their positions at the center, inner corner, and outer corner of the SD, respectively. (*B*) A representative insulating differential conductance (d*I*/d*V*) spectrum acquired on a vacuum-cleaved 1*T*-TaSe_2_ sample. (*C*) The topography of a singe-step region with moiré pattern in 1*T*-TaSe_2_ ($V_{b}=-600$ mV, $I_{t}=20$ pA). White and black arrows denote the lattice directions of the SD supercells below and above the step, respectively. (*D*) Fourier transforms of topographic images for the upper and lower surfaces. The white, blue, and yellow circles, along with their corresponding arrows, respectively denote the Bragg peaks in reciprocal space associated with the lower SD superlattice, the upper SD superlattice, and the upper moiré superlattice (atomic moiré).

Monolayer 1*T*-TaSe_2_ stands apart from weakly correlated 2D materials, garnering significant attention due to its pronounced strong correlation effects (11–15). At low temperatures, electron–phonon interactions drive lattice distortions that yield a commensurate $\sqrt{13}\times \sqrt{13}$ charge density wave (CDW) phase, characterized by a star of David (SD) superlattice as shown in Fig. 1*A*. Within each SD supercell, 13 Ta atoms collectively contribute one half-filled electronic band. Remarkably, despite this half-filled configuration, molecular beam epitaxy (MBE)-grown monolayers exhibit a clear insulating ground state (11, 14), supporting its characterization as a Mott–Hubbard insulator where strong Coulomb interactions dominate over kinetic energy (16, 17). The CDW-induced flat band is considered the primary origin of these strong correlation effects. This behavior shares parallels with isostructural monolayers: 1*T*-TaS_2_ (14, 18, 19) and 1*T*-NbSe_2_ both develop similar CDW superlattices and exhibit strongly correlated insulating gaps (20, 21).

The presence of a strongly correlated monolayer inherently complicates interactions within multilayer vdW systems. For instance, alternating stacking of single-layer insulating 1*T*-TaS_2_ and metallic 1*H*-TaS_2_ allows the localized spins in the 1*T* layer to interact with the itinerant electrons of the 1*H* layer, potentially inducing a Kondo effect (22, 23). However, the manifestation of this effect varies significantly across different samples (22–26), likely influenced by charge transfer and subtle differences in interlayer spacing (27). Intriguingly, while both 1*T*-TaS_2_ and 1*T*-TaSe_2_ monolayers share similar CDW superlattice structures and Mott insulating behavior, their bulk phases exhibit contrasting transport behaviors: insulating for 1*T*-TaS_2_ (28) and metallic for 1*T*-TaSe_2_ (29). Microscopically, both insulating and metallic states have been observed on the surface of bulk 1*T*-TaSe_2_, and the stacking order effect is a possible tuning parameter (29, 30). In 1*T*-TaS_2_, a small-gap insulating state has been correlated with AC stacking, proposed as the signature of the intrinsic Mott gap (31). Although statistical analyses of stacking configurations and their effects have been conducted for both materials, the limited number of stacking geometries hinders the establish of a comprehensive mechanism (28, 29). There is much debate over the interpretation of various electronic phases in multilayered 1*T*-TaSe_2_ and 1*T*-TaS_2_ (28–37).

In this work, scanning tunneling microscopy (STM) imaging of bulk 1*T*-TaSe_2_ reveals a twisted bilayer structure (<4^°^) formed near a surface step. Near the step edge, we observe a rotated SD superlattice on the upper terrace and a corresponding moiré modulation of the SD intensity in the topographic image. Spatially resolved scanning tunneling spectroscopy (STS) further uncovers a continuous insulator-to-metal transition, manifested as a gradual closing of the low-energy gap near the Fermi level. Notably, the period of this gap modulation differs from that of the topographic moiré pattern. Numerical simulations indicate that the topographic moiré originates from the twisted atomic lattice, while the gap modulation arises solely from the moiré pattern of the SD superlattice.

Combining simulations and density functional theory (DFT), we show that twist-induced changes in the SD stacking configuration fundamentally modify the bilayer hybridization—a key mechanism governing the low-energy electronic evolution—though the inclusion of an electronic correlation parameter U remains essential for accurate bilayer DFT+U calculations. Even in the least hybridized regions of the twisted bilayer, the monolayer Mott insulating state is completely suppressed, unlike in strongly decoupled monolayers. These findings establish the twisted bilayer of strongly correlated 1*T*-TaSe_2_ as a model system exhibiting two concurrent moiré patterns with distinct electronic tuning effects. The presence of such a twisted bilayer at the surface is attributed to its decoupling from the underlying layers.

Finally, we use the moiré-period gap map as the interlayer interaction potential V(r). Fourier transformation yields discrete coefficients $V_{G}$, which form the basis of a continuum model for the momentum-space band structure of the twisted system. We find that $V_{G}$ drives multiple round-trip scattering processes of electrons between the layers, ultimately generating numerous pairs of split flat bands with distinct energy gaps. Altogether, we observe and characterize the twisted bilayer composed of a strongly correlated monolayer constituent (38), providing a model system that exhibits concurrent dual moiré patterns exerting distinct tuning effects. The construction of a low-energy continuum model also elucidates the moiré bands of such a twisted flat-band system.

## Results

The single-crystal 1*T*-TaSe_2_ studied here was synthesized via chemical vapor transport (CVT); details of growth and STM instrumentation are provided in *Materials and Methods*. Monolayer 1*T*-TaSe_2_’s half-filled SD superlattice is generally understood as a Mott–Hubbard insulator (11). Despite bulk 1*T*-TaSe_2_ exhibiting metallic transport behavior, STM reveals both insulating and metallic states on its surface. Fig. 1*B* shows a representative differential conductance (d*I*/d*V*) spectrum measured on cleaved bulk 1*T*-TaSe_2_, characterized as a large-gap insulating state. This spectrum exhibits two distinct peaks located at approximately ±Δ relative to the Fermi energy (zero bias). The central gap region spans 2Δ around the Fermi level. Although a small nonzero conductance persists within the gap, this spectrum in bulk 1*T*-TaSe_2_ has been referred to as a large-gap insulating state with a gap size Δ≈220 mV (29).

Fig. 1*C* shows a topographic image T(r) of a step on 1*T*-TaSe_2_. The lower terrace exhibits the SD superlattice and a large insulating gap, while the upper terrace shows an additional moiré pattern, indicating interlayer twisting. Fourier transform (FT) analysis in Fig. 1*D* confirms this: The FT of the lower terrace shows only the SD Bragg peaks (white arrows), whereas the upper terrace’s FT reveals both SD peaks (blue arrows) and additional moiré peaks (yellow arrows). The relative positions of these peaks determine a twist angle of $3.9^{{}^{\circ}}$ between the terraces, with a $14^{{}^{\circ}}$ angle and 0.26 amplitude ratio between the moiré and SD vectors. The relationship between these two experimental values and the twist angle will be discussed in detail later.

**Fig. 2.**
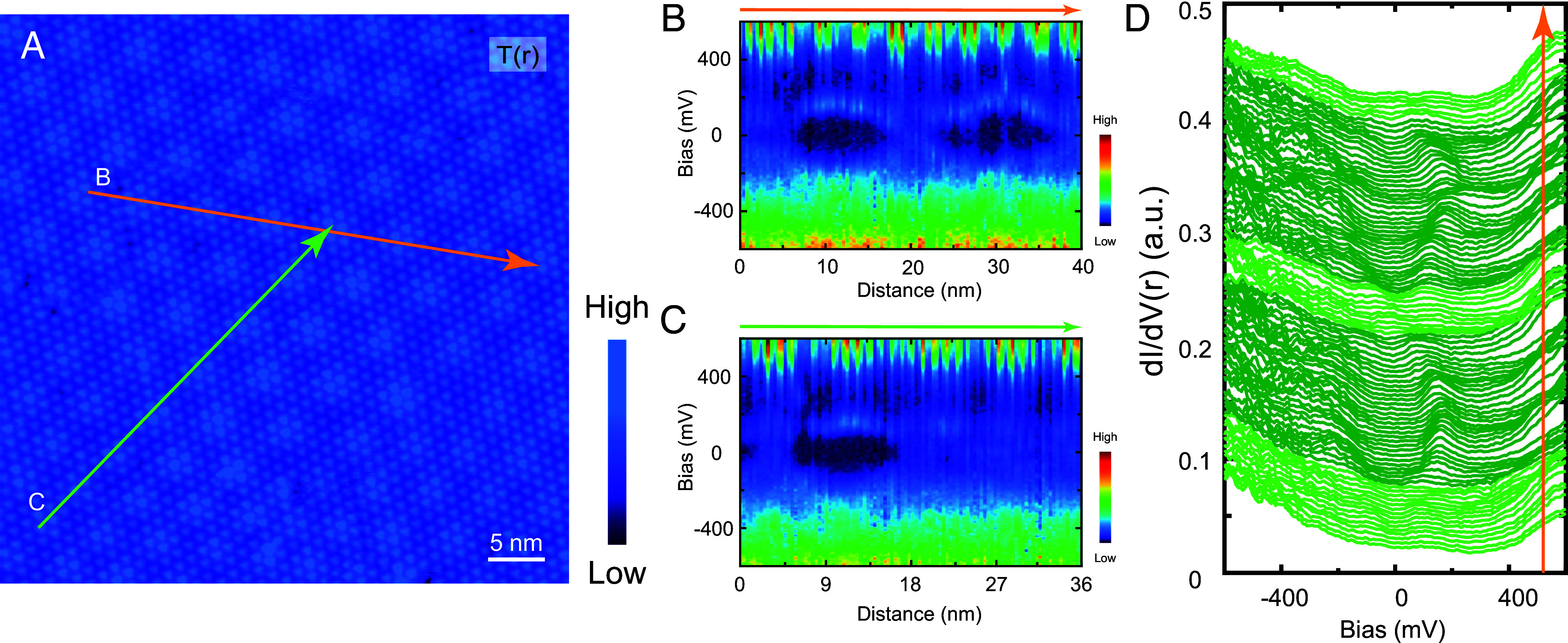
(*A*) Constant-current 50 nm × 50 nm STM topography of the SD moiré pattern on the upper surface in the step region shown in Fig. 1*A* ($V_{b}=-600$ mV, $I_{t}=20$ pA). (*B* and *C*) Spatially resolved d*I*/d*V* spectra along the orange and green arrows in (*A*), respectively. (*D*) Waterfall plot of the d*I*/d*V* spectra along the orange arrow in (*A*). The dark green and light green spectra correspond to gapped and metallic electronic states, respectively.

Fig. 2*A* shows an enlarged moiré topography with d*I*/d*V* spectra acquired along a line crossing the moiré maxima (orange line). The spectra in Fig. 2 *B* and *D* reveal a continuous metal-to-insulator transition across the moiré, forming “eye-shaped” regions of suppressed DOS at low energies, alongside a topographically aligned high-energy DOS peak at +600 mV attributed to the CDW band (39). For comparison, spectra along a different path (green line, Fig. 2*C*) show a similar “eye-shaped” gapped region on the left side. However, no such gapped region is observed on the right side, or the region on the right remains metallic.

The direct link between the moiré pattern and the variable gap in d*I*/d*V* spectra was initially unclear. We hypothesized that the observed moiré arises from a rigid twist of the entire upper layer (atomic and SD lattices together). This model is confirmed by comparing theoretical and experimental values (*SI Appendix*, section S1): For a $3.9^{{}^{\circ}}$ interlayer twist, the calculated relative angle ($14.15^{{}^{\circ}}$) and amplitude ratio (0.25) between the moiré and SD Bragg vectors agree well with the measured $14^{{}^{\circ}}$ (with $\pm 2^{{}^{\circ}}$ uncertainty brought by a single pixel) and 0.26. This confirms the moiré pattern stems from twisted atomic lattices, which modulate high-energy CDW features (e.g., the +600 mV peak) in the topography. Unlike other systems involving strain or reconstruction (40), the modulation here is purely twist-induced.

**Fig. 3.**
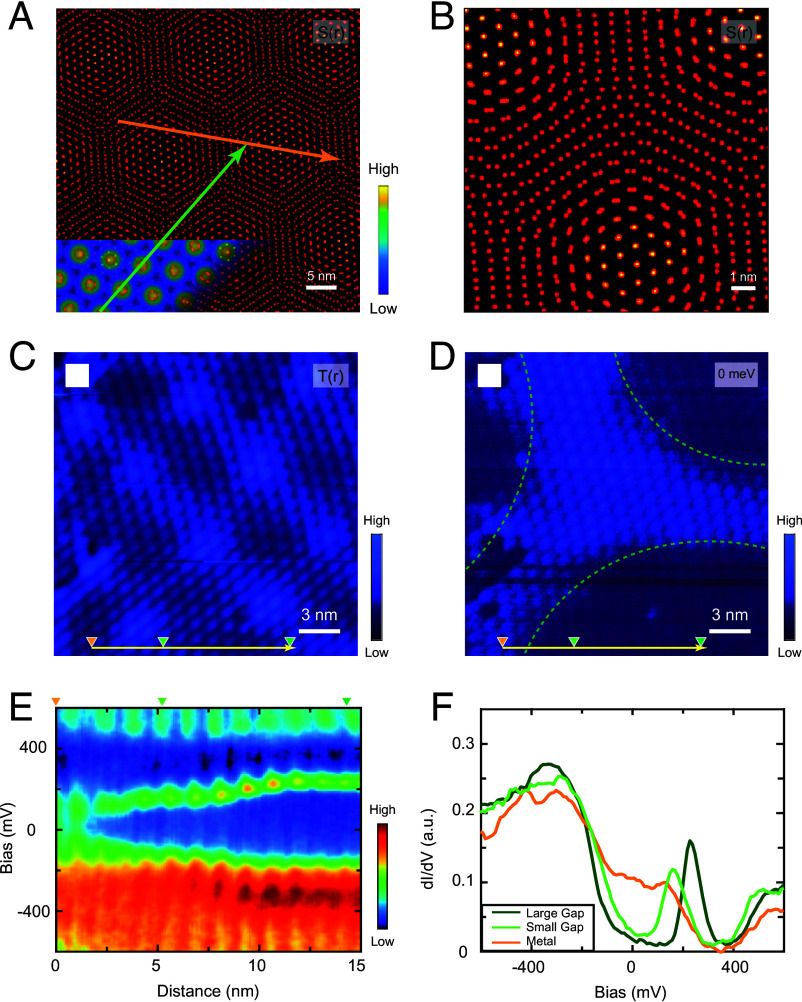
(*A*) Simulation of two SD superlattices with a twist angle of ∼3.9^°^ (50 nm × 50 nm). The orange and green lines indicate the positions of the two line cuts shown in Fig. 2*A*. (*B*) An enlarged view of a region from (*A*), highlighting the misalignment between the upper and lower layers of the SD superlattices. (*C*) Constant-current STM topography (22 nm × 22 nm) of another SD moiré pattern with a twist angle of ∼2.0^°^ ($V_{b}=-600$ mV, $I_{t}=200$ pA). (*D*) Simultaneously acquired zero-bias d*I*/d*V* map corresponding to (*C*). Green dashed lines approximately outline the metallic region with enhanced density of states (DOS). (*E*) Distance-dependent d*I*/d*V* spectra acquired along the yellow arrow in (*C* and *D*). (*F*) Three representative d*I*/d*V* spectra extracted at different locations marked in (*C* and *D*).

Our data reveal that the tuning of the low-energy peaks and gap size cannot be explained by the atomic moiré structure. Consequently, we investigate the distinct moiré pattern arising from the twisted SD superlattice itself. Fig. 3*A* directly visualizes this SD moiré pattern, characterized by a spatial period $\sqrt{13}$ times longer than the atomic moiré (superimposed on the up-left corner) and a $13.9^{{}^{\circ}}$ orientation difference relative to it. Crucially, correlating the positions of the STS line scans (orange and green lines) with the SD moiré reveals a direct link: The orange line traverses two SD moiré maxima, corresponding to its two gap tuning cycles, while half the green line passes near an SD maximum (yielding gapped spectra) and the other half crosses an SD minimum (resulting in metallic spectra). This precise spatial correspondence demonstrates that the low-energy gap modulation is governed by the SD superlattice moiré, not the atomic-scale moiré.

The generality of the phenomenon is confirmed by data from a second sample (*SI Appendix*, sections S2 and S3), which exhibits a moiré pattern arising from a ∼2^°^ twisted bilayer in Fig. 3*C*. A zero-bias d*I*/d*V* map in Fig. 3*D* reveals a *Y*-shaped metallic feature centered on the SD moiré minima, contrasting with gapped regions at the maxima. Spectra acquired along the line indicated by the yellow arrow in Fig. 3*C* show a continuous transition from a metallic state to a large-gap insulating state, as displayed in Fig. 3*E*. Three representative spectra in Fig. 3*F* show the sequence from a metallic state, through a small-gap intermediate, to a large-gap state characterized by a +220 mV peak. This evolution shows a direct connection between the SD moiré position and the low-energy electronic states.

In the schematic representation of the SD moiré pattern shown in Fig. 3 *A* and *B*, the SDs are depicted at a reduced scale to clearly illustrate the relative positions of the upper and lower SDs within the twisted structure. At the SD moiré maxima, upper and lower SDs align vertically, forming AA stacking. Moving away from these maxima, the horizontal displacement between upper and lower SDs gradually increases. Upon reaching the SD moiré minima, the stacking configuration is characterized by significant horizontal shifts combined with relative rotations. Critically, our spectroscopic results demonstrate that this continuous variation in stacking geometry directly correlates with the electronic state: The large-gap insulating spectra occur precisely at the SD moiré maxima, while the metallic spectra emerge at the SD moiré minima.

**Fig. 4.**
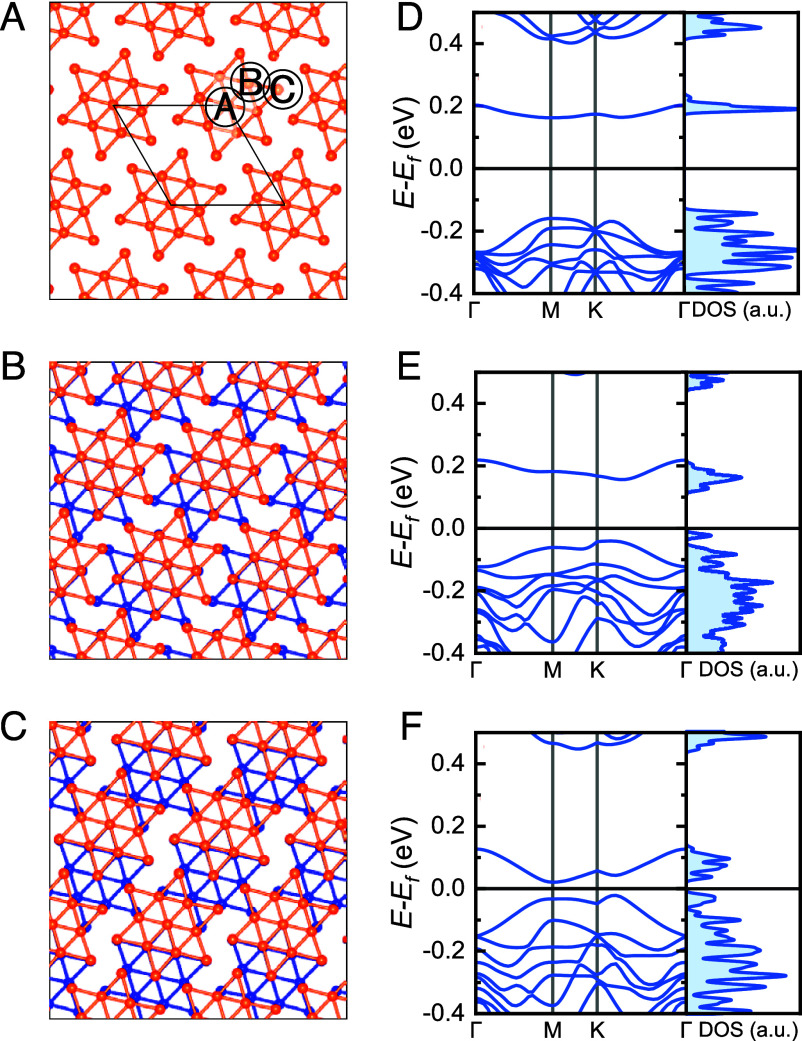
(*A*–*C*) Schematic illustrations of bilayer 1*T*-TaSe_2_ with AA-, AB-, and AC- stacking configurations, respectively, where only the Ta atoms are shown to highlight the SD supercell structure. The orange and blue spheres represent Ta atoms in different layers. (*D*–*F*) DFT+U band structures (U = 2 eV) and density of states (DOS) for bilayer 1*T*-TaSe_2_ with AA-, AB-, and AC-stacking orders, respectively.

DFT calculations for a twisted bilayer 1*T*-TaSe_2_ model three stacking configurations (AA, AB, AC in Fig. 4*A*–*C*), approximating key regions of the SD moiré pattern (for details on DFT, see *Materials and Methods*). The maximal inter-SD horizontal separation (∼6.0 Å) at moiré minima closely matches the AC stacking displacement (*SI Appendix*, Fig. S4). Using DFT+U (Hubbard U correction) with vdW corrections (optB88-vdW functional) (41, 42), we find that the coupled bilayer forms two separate flat bands, with the bandgap decreasing substantially from AA (0.322 eV) to AB (0.197 eV) and further to AC (0.042 eV) stacking. The AA and AB stackings can be assigned as a large-gap and an intermediate small-gap state in this process. Although a very small gap remains in the AC stacking configuration, this pronounced reduction trend in bandgap is in direct agreement with experimental observations. Because the coupled bilayer forms two separate flat bands, the gap peaks and sizes observed in STS measurements are most appropriately compared to the calculated integrated DOS (*Right* panels of Fig. 4*D*–*F*). The AC stacking configuration exhibits a very small gap in DFT calculations, and the discrepancy between this result and the experimentally observed metallic behavior may be attributed to the weak coupling between the bilayer and the underlying bulk material.

At the center of the SD moiré pattern—where the top and bottom SD units adopt AA stacking—both experiment and DFT reveal a large-gap insulating state. Although this gap resembles the monolayer Mott insulating state, DFT calculations confirm it is distinct from the Hubbard bands obtained in spin-polarized calculations (*SI Appendix*, section S5). In fact, interlayer hybridization at AA-stacked sites reconstructs the two monolayer Mott insulators into a band insulator, though electronic correlations (parameter U) remain needed (*SI Appendix*, section S4 and Fig. S6). For simplicity, we still refer to this hybridization-induced gap as a large-gap spectrum.

Unlike conventional parabolic bands, the bilayer exhibits flat bands both above and below the Fermi level. These flat bands arise from a real-space bilayer interaction Hamiltonian $H_{\mathrm{in}}=t_{⊥}\sum_{R}\left(c_{R,1}^{†}c_{R,2}+h.c.\right)$, where electrons hop between the same supersite *R* in the layer 1 and layer 2 under AA stacking, and h.c. denotes the Hermitian conjugate, representing the reverse hopping process. The Bloch theorem for periodic lattices enables the transformation of the electron operator from real space to momentum space, allowing the Hamiltonian to be expressed in momentum space as $H_{\mathrm{in}}=t_{⊥}\sum_{k}\left(c_{k,1}^{†}c_{k,2}+h.c.\right)$. In this representation, the Hamiltonian in momentum space has an extra off-diagonal matrix element of magnitude $t_{⊥}$ for each *k*-point. Diagonalization of the Hamiltonian leads to the splitting of the degenerate flat band into two distinct flat bands. This flat-band character accounts for the pronounced experimental spectral peak at +220 mV. Then the hopping amplitude $t_{⊥}$ is equivalent to the gap size Δ. The energy separation between this flat band and higher-energy bands also aligns with the conductance dip observed near +400 mV in Fig. 3*F*. Notably, the valence flat band below the Fermi level—unlike the case in bilayer 1*T*-TaS_2_ (27)—mixes with lower-lying bands, leading to a broader spectral feature below −220 mV compared to the sharper +220 mV peak.

Moving away from the moiré center, increased horizontal displacement between SD layers (reaching AC-like horizontal separation near 6 Å, *SI Appendix*, section S4) progressively weakens interlayer hybridization. The gap decreases as the hybridization strength is reduced, while the flat-band character is retained. Although weakened hybridization drives the system toward a metallic state near the AC region, the monolayer Mott insulator does not reappear due to residual interlayer coupling—explaining the continuous suppression of the monolayer Mott state across the moiré landscape.

Due to the mixing of the valence flat band with other bands, we extract the local gap Δ from the positive spectral peak rather than the negative peak. From the d*I*/d*V* spectra acquired across the region shown in Fig. 3*C*, the local gap Δ(r) is determined at each spatial point (220×220 pixels), enabling the construction of a real-space gap map in which metallic regions are assigned a zero gap. As shown in *SI Appendix*, Fig. S7, the resulting gap map Δ(r) displays a central *Y*-shaped region, consistent with the feature observed in Fig. 3*D*.

While the real-space electronic modulation by the moiré potential has been demonstrated, understanding the momentum-space moiré band structure remains a key challenge. Leveraging the flat-band nature and interlayer hybridization in 1*T*-TaSe_2_, we adopt the gap map Δ(r) as a representation of the interlayer interaction potential V(r). Owing to the finite scan area, a periodically extended V(r) is constructed via symmetry-based replication (*SI Appendix*, section S6 and Fig. S7).

The periodicity of V(r) with the SD moiré lattice leads to a discrete Fourier transform V(q), which is nonzero only at reciprocal lattice vectors $G=mb_{M}^{1}+nb_{M}^{2}$, where *m*, *n* are integers and $b_{M}^{i}$ (i=1,2) are moiré reciprocal basis vectors. We obtain $V_{G_{0}}=0.1564$ eV and $V_{G_{i}}=0.0259$ eV (i=1,2,3; *SI Appendix*, Fig. S7), and incorporate these coefficients into a continuum model for twisted 1*T*-TaSe_2_ (10, 43, 44).

Even with only two representative cases, our model captures the essential physics: The monolayer flat band facilitates higher-order interlayer scattering via $V_{G}$, successively coupling a state at k to k+G, then to $k+\;G\;+\;G^{\prime }$, etc., ultimately generating multiple pairs of split flat bands with different energy gaps (*SI Appendix*, section S6 and Fig. S8). Each eigenstate corresponds to a distinct superposition of these momentum-shifted components.

## Discussion

While atomic stacking universally governs electronic hybridization in vdW homojunctions (maximized at AA registry and progressively weakened by increasing displacement to create an atomic moiré potential), bilayer 1*T*-TaSe_2_ exhibits a critical distinction. Here, the SD moiré pattern coexists with the atomic moiré, yet it is the SD moiré period that precisely dictates the moiré-modulated low-energy gap. This unique behavior arises because the commensurate CDW phase of 1*T*-TaSe_2_ possesses an orbital texture localized within each SD superlattice (39, 45). Consequently, the low-energy electronic physics is fundamentally dominated by the stacking configuration between the upper and lower SD superlattices, rather than the relative alignment of the underlying atomic lattices.

While DFT shows that bilayer stacking order critically modulates the electronic state, interpreting the moiré modulation on bulk surfaces is more complex. The measured surface spectrum results from a competition: the interaction between the top two layers versus their coupling to the substrate. When the top bilayer interaction dominates, its characteristic electronic properties become observable. This explains why metastable, bilayer-like states appear near step edges, where constrained relaxation favors specific stackings (28, 29). Conversely, if the surface layer is strongly decoupled from the bulk, it can exhibit the signature of an isolated monolayer. This framework suggests that bulk metallicity in 1*T*-TaSe_2_ arises from the hybridization of Mott-insulating layers, and our initial interpretation of the large-gap state solely as a monolayer characteristic was incomplete (29), as bilayer AA stacking can produce a similar large-gap band insulator (27).

The observed twist regions within the atomic lattice are unlikely to originate from the single crystal growth process. Instead, these localized rotational misalignments likely arise from transient, microscopic-scale torque generated during mechanical cleavage—specifically, the rapid tapping used to separate the sample. This induced torque can drive lattice rotations confined to specific areas on the cleaved surface. The occurrence of these twisted regions on any given cleaved surface remains a low-probability event, representing a rare phenomenon observed among a large number of cleaved samples with single-step surfaces (29). Prior to the development of in situ twist-controllable graphene devices, experimental evidence for moiré-induced flat bands initially emerged from naturally occurring twisted graphene domains on highly oriented pyrolytic graphite (HOPG) surfaces (46). Similarly, our experiments confirmed the existence of twisted bilayer 1*T*-TaSe_2_ as a stable state without atomic reconstructions and domain networks, based on accidental discoveries, and included a detailed investigation on its electronic structure. Although technically hard, the development of controllable twisted devices could offer opportunities for probing and manipulating correlated electronic phases in such systems (47).

Our study introduces a prototypical moiré system composed of strongly correlated flat-band monolayers. In real space, split flat bands with distinct energy gaps are observed, corresponding to different interlayer stacking orders. In momentum space, the monolayer flat band enables multiple scattering and coupling among momentum-shifted states via the moiré potential, producing numerous split flat bands with varying gap magnitudes. Band crossings between these coupled states further promote the formation of superflat bands after hybridization and splitting—a phenomenon deserving further investigation under ultralow-temperature conditions. Although interlayer hybridization in the twisted system substantially suppresses correlation effects, hole doping of the split flat bands can induce a correlated electronic state reminiscent of that in a doped half-filled Mott insulator. Together, these findings provide insights and a deeper understanding of the electronic behavior in twisted 1*T*-TaSe_2_ and related multilayer materials.

## Materials and Methods

### Material Preparation.

Single-crystal 1*T*-TaSe_2_ was synthesized by the chemical vapor transport (CVT) method using iodine as the transport agent. A mixture of Ta (99.99%) and Se (99.99%) powders with a nominal molar ratio 1:2, along with I_2_ (0.15 g), was sealed in an evacuated quartz tube and heated in a two-zone furnace at 1,000 ^°^C (source zone) and 900 ^°^C (growth zone) for 10 d. The resulting crystals were quenched in ice water, and their stoichiometry was verified by X-ray energy dispersive spectroscopy (EDS).

### STM Experiment.

STM measurements were performed using an ultrahigh-vacuum, low-temperature system. Single-crystal 1*T*-TaSe_2_ samples were cleaved in situ, and all data were acquired at 77 K, where the sample is in the commensurate charge density wave (CCDW) phase. Topographic images were obtained with a sample bias of $V_{b}=-600$ mV and tunneling currents of $I_{t}=20$ pA or $I_{t}=200$ pA. Differential conductance (d*I*/d*V*) spectra were recorded with a lock-in amplifier using a 10 mV modulation at 1,213.7 Hz. Tungsten tips were electrochemically etched and conditioned on Au(111).

### DFT Calculation.

DFT calculations were performed using the Vienna ab initio simulation package (VASP) (48) with projector-augmented wave (PAW) pseudopotentials and the Perdew–Burke–Ernzerhof (PBE) exchange-correlation functional (49–51). Van der Waals interactions were accounted for using the vdW-DF method (optB88-vdW) (42), and on-site Coulomb interactions were included via the DFT+U approach (41). Further calculation details and discussions on spin polarization are provided in *SI Appendix*, section S5.

### Model Simulation.

A continuum model based on a triangular lattice of SD clusters is established for twisted bilayer 1*T*-TaSe_2_. The intralayer Hamiltonian is derived by fitting DFT results using tight-binding methods. Interlayer coupling strengths are extracted from the Fourier transform of the gap map. Further details are provided in *SI Appendix*, section S6.

## Supplementary Material

Appendix 01 (PDF)

## Data Availability

Experimental data and codes have been deposited in Zenodo repository https://doi.org/10.5281/zenodo.17518390 (52).
