# Supplementary material for: Modulation of electronic structure via dual moiré patterns in twisted 1T-TaSe2
Source: Proc Natl Acad Sci U S A. 2026 Mar 11;123(11):e2520703123. doi: 10.1073/pnas.2520703123 (PMC12994195; doi:10.1073/pnas.2520703123)
Supplement: Supplementary file 1 — Appendix 01 (PDF) [file pnas.2520703123.sapp.pdf]

Supporting Information for Modulation of electronic structure via dual moiré patterns in twisted $1T$-$TaSe_2$

Yonghao Liu[1,†], Yuan Zheng[1,†], Kun Yang[2,†], Wenhao Zhang[1], Zongxiu Wu[1], Jingjing Gao[3], Xuan Luo[3], Yuping Sun[3,4,5,*], Jin Zhang[2,*], and Yi Yin[1,5,*]

[1] *Zhejiang Key Laboratory of Micro-Nano Quantum Chips and Quantum Control, School of Physics, Zhejiang University, Hangzhou 310027, China*

[2] *Laboratory of Theoretical and Computational Nanoscience, National Center for Nanoscience and Technology, Chinese Academy of Sciences, Beijing 100190, China*

[3] *Key Laboratory of Materials Physics, Institute of Solid-State Physics, HFIPS Chinese Academy of Sciences, Hefei 230031, China*

[4] *High Magnetic Field Laboratory, HFIPS Chinese Academy of Sciences, Hefei 230031, China*

[5] *Collaborative Innovation Center of Advanced Microstructures, Nanjing University, Nanjing 210093, China*

January 4, 2026

This PDF file includes:

- Section S1, Fig. S1
- Section S2, Fig. S2
- Section S3, Fig. S3
- Section S4, Fig. S4

- Section S5, Fig. S5, Fig. S6
- Section S6, Table S1, Fig. S7, Fig. S8
- SI References

Section S1

Moiré patterns formed in twisted $1T$-$TaSe_2$

As shown in Fig. 2 of the main text, a unique single-step region in $1T$-$TaSe_2$ exhibits a twisted structure that likely formed accidentally during cleaving. A distinct periodic pattern—distinct from the star of David (SD) superlattice—is observed on the upper terrace. We attribute this pattern to a moiré interference arising from rotated atomic lattices. Although the moiré pattern from twisted SD superlattices is nearly invisible in the topography, its presence can be inferred from theoretical simulations. Since both the atomic and SD superlattices are triangular with similar symmetry but different lattice constants and orientations, we begin by analyzing the moiré pattern using ideal triangular lattices as a model system.

Figure S1 **a** and **b** show two identical ideal triangular lattices, with **b** rotated counterclockwise by 10° relative to **a**. The resulting moiré pattern, shown in Fig. S1 **c**, is the sum of the simulation data of two lattices. Figure S1 **e** displays the two-dimensional Fourier transform of **c**, revealing its reciprocal-space features. The Fourier transform of the triangular lattice exhibits six primary Bragg peaks, corresponding to its reciprocal lattice vectors and reflecting the periodicity of the real-space lattice. In Fig. S1 **e**, the yellow and green arrows mark the Bragg peaks associated with the reciprocal vectors $\mathbf{b}_1$ (bottom layer) and $\mathbf{b}_2$ (top layer), respectively, which are rotated by 10° relative to each other.

Figure S1 **d** presents the moiré modulation extracted from the product of the simulation data of two lattices, followed by reciprocal-space Gaussian low-pass filtering (filter radius $\sigma = 3.2\,a_0$, where a_0 is the lattice constant). The moiré maxima—defined as positions where top and bottom atoms align vertically—appear as red regions in **d**, while the minima, corresponding to complete atomic mismatch, are shown in blue.

In each layer of $1T$-$TaSe_2$, two triangular lattices coexist: the atomic lattice and the CDW lattice. A relative twist between layers thus generates two distinct moiré patterns—one from the atomic lattices and one from the CDW superlattices. The maxima of each moiré pattern correspond to regions where the respective lattices are perfectly aligned, while the minima occur where they are maximally mismatched.

The Fourier transform of Fig. S1 **d** is shown in Fig. S1 **f**, where a set of low-amplitude Bragg peaks reflects the periodic moiré modulation. The vectors marked by magenta and cyan arrows in **f**—identical to those in **e**—correspond to the difference $\mathbf{b}_\mathrm{M} = \mathbf{b}_2 - \mathbf{b}_1$ between the green- and yellow-marked vectors. Notably, these small-amplitude vectors are absent near the origin in Fig. S1 **e**.

Thus, the moiré pattern can be interpreted as a two-dimensional beat phenomenon. Geometric analysis shows that the angle between the green and magenta vectors is 85°. For comparison, a green vector is reproduced in Fig. S1 **f**. Given the 60° angle between the magenta and cyan vectors, the angle between the green and cyan vectors is 25°, indicating that the moiré pattern is rotated counterclockwise by 25° relative to the rotated triangular lattice (defined by $\mathbf{b}_2$). The angular relationships are also labeled in Fig. S1 **c**. The magnitude ratio between the magenta and green vectors is $2\sin(5°)$. From the moiré pattern of ideal triangular lattices, the following relationship can be derived:

$$|\mathbf{b}_\mathrm{M}| = 2|\mathbf{b}_2|\sin(\theta/2), \tag{1}$$

$$\alpha = \arccos(|\mathbf{b}_\mathrm{M} \cdot \mathbf{b}_2|/|\mathbf{b}_\mathrm{M}||\mathbf{b}_2|) = (\pi - \theta)/2, \tag{2}$$

in which θ is the twist angle, $\mathbf{b}_\mathrm{M}$ and $\mathbf{b}_2$ are the Bragg peak vectors of the moiré lattice and the rotated lattice (layer 2) in reciprocal space, respectively, and α is the angle between $\mathbf{b}_\mathrm{M}$ and $\mathbf{b}_2$. For $\theta = 3.9°$, $\alpha = 88.05°$. Subtracting 60° and the 13.9° angle between the SD superlattice and atomic lattice vectors yields a theoretical angle of 14.15° between the atomic moiré vector and the top-layer SD vector in Fig. 1(d), in good agreement with the experimental value around 14° (with ±2° uncertainty brought by a single pixel). The ratio of the atomic moiré vector to the atomic lattice vector is given by $2\sin(3.9°/2)$. Considering that the SD superlattice period is $\sqrt{13}$ times larger, the ratio between the atomic moiré vector and the SD superlattice vector is calculated as $2\sin(3.9°/2) \times \sqrt{13} = 0.25$, which is in close agreement with the experimentally measured value of 0.26.

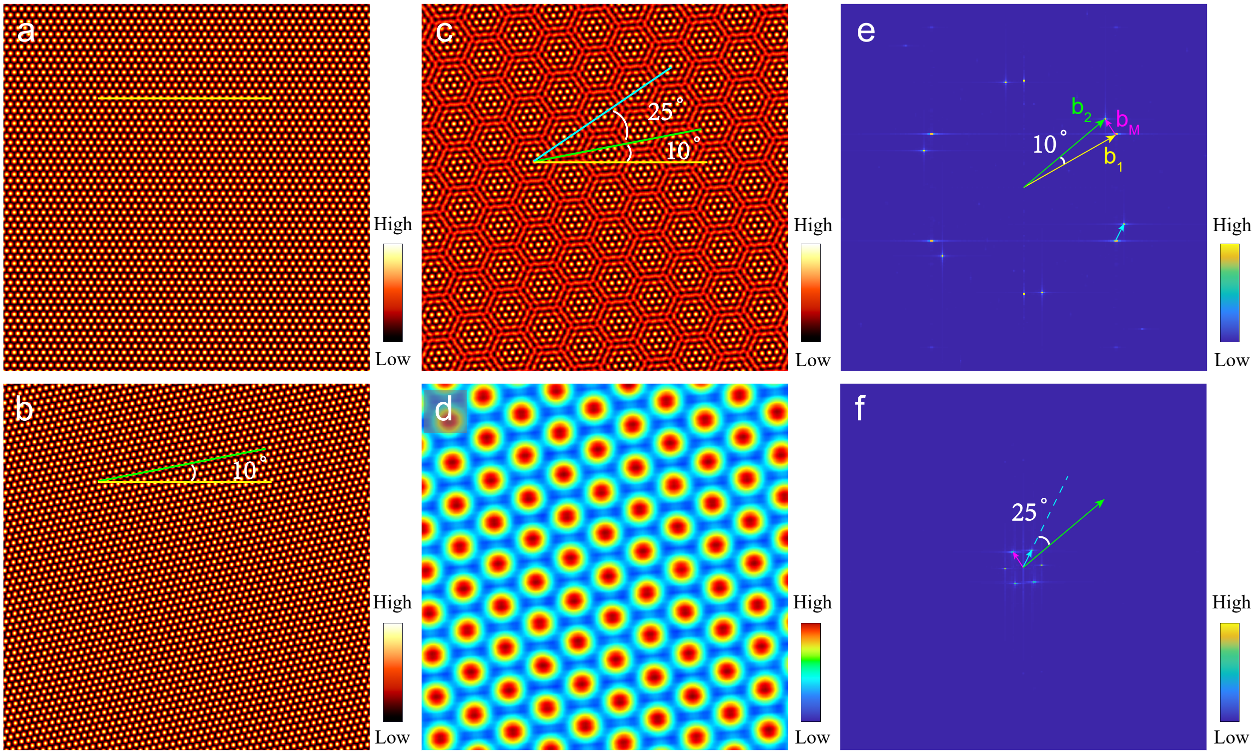

Fig. S1. **a**, An ideal triangular lattice. **b**, A second, top layer lattice rotated counterclockwise by 10°. **c**, The addition of the two triangular lattices shown in **a** and **b**, generating a moiré pattern with a longer spatial period. **d**, The moiré pattern obtained by multiplying **a** and **b** and then performing 2D low-pass filtering. **e** and **f** show the Fourier transforms of **c** and **d**, respectively.

Section S2

Extended topography of the moiré region

Figure S2 **a** presents an extended topographic view that extends beyond the scope of Fig. 3*C*. To further elucidate the characteristics of this moiré region, supplementary markers have been introduced in Fig. S2 **b**. The topography above the step can be divided into three distinct regions: normal region, moiré region 1 and moiré region 2.

The red dashed lines in the top-left corner, together with the edge of the step, outline a normal region without any atomic twist. Another normal region is observed on the right side of the topography, where the boundary is a domain wall in the SD superlattice that traverses several defects. On the left side, the yellow dashed lines, together with the left edge of the central white square box, enclose a small twisted area referred to as moiré region 1. This region exhibits a small moiré period (atomic moiré pattern) of approximately 4.7 nm under a twist angle of 3.1°. The remaining region between moiré region 1 and the right normal region, designated as moiré region 2, demonstrates a larger moiré period (atomic moiré pattern) of approximately 10.7 nm under a slightly smaller twist angle of 2.0°. The central white square frame is the area of Fig. 3*C* and *D* in the main text, and the solid white square in the upper left corner indicates the orientation of this area.

The abundance of topographic features on the step can be attributed to the presence of defects and domain walls. It is evident that the boundaries between normal regions and twisted regions intersect with defects and domain walls, potentially inducing inhomogeneous rotation during the cleaving process to achieve a stable state. The large crack extending from the step edge may also promote atomic twisting, resulting in distinct rotation angles between moiré region 1 and moiré region 2.

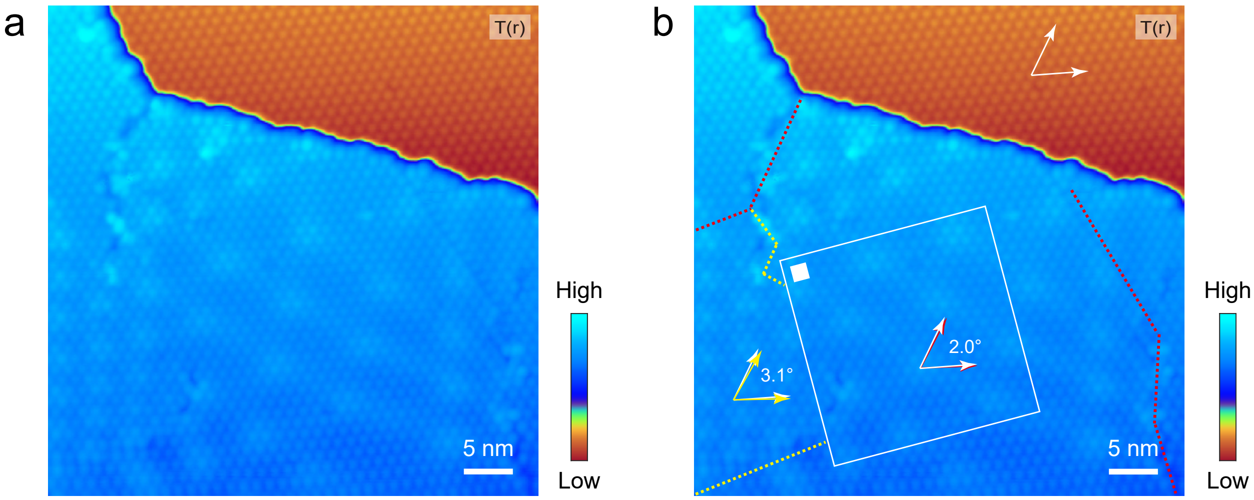

Fig. S2. **a**, Constant-current topography ($50\,\mathrm{nm} \times 50\,\mathrm{nm}$, $V_\mathrm{b} = -1\mathrm{V}$, $I_\mathrm{t} = 20\mathrm{pA}$) of the region shown in Fig. 3*C* and *D*. **b**, The same topography with annotated highlights indicating the details of the moiré pattern. The white square marks the direction of the area shown in Fig. 3*C* and *D*.

Section S3

Distance-dependent dI/dV spectra above and below the step

We obtained a series of distance-dependent dI/dV spectra along the red arrowed line in Fig. S3 **a**. Figure S3 **b** shows the height profile along the red arrowed line, with the step height being approximately 6.4 Å. A typical large-gap insulating spectrum is presented in Fig. S3 **c**, measured at the position indicated by the green triangle in Fig. S3 **a**. The distance-dependent dI/dV spectra depicted in Fig. S3 **d** reveal a continuous metal-insulator transition above the step and a constant large-gap insulating state below the step. The spectral characteristics of this linecut are consistent with the zero-bias map shown in Fig. S3 **a**, further validating the theoretical simulations described in the main text.

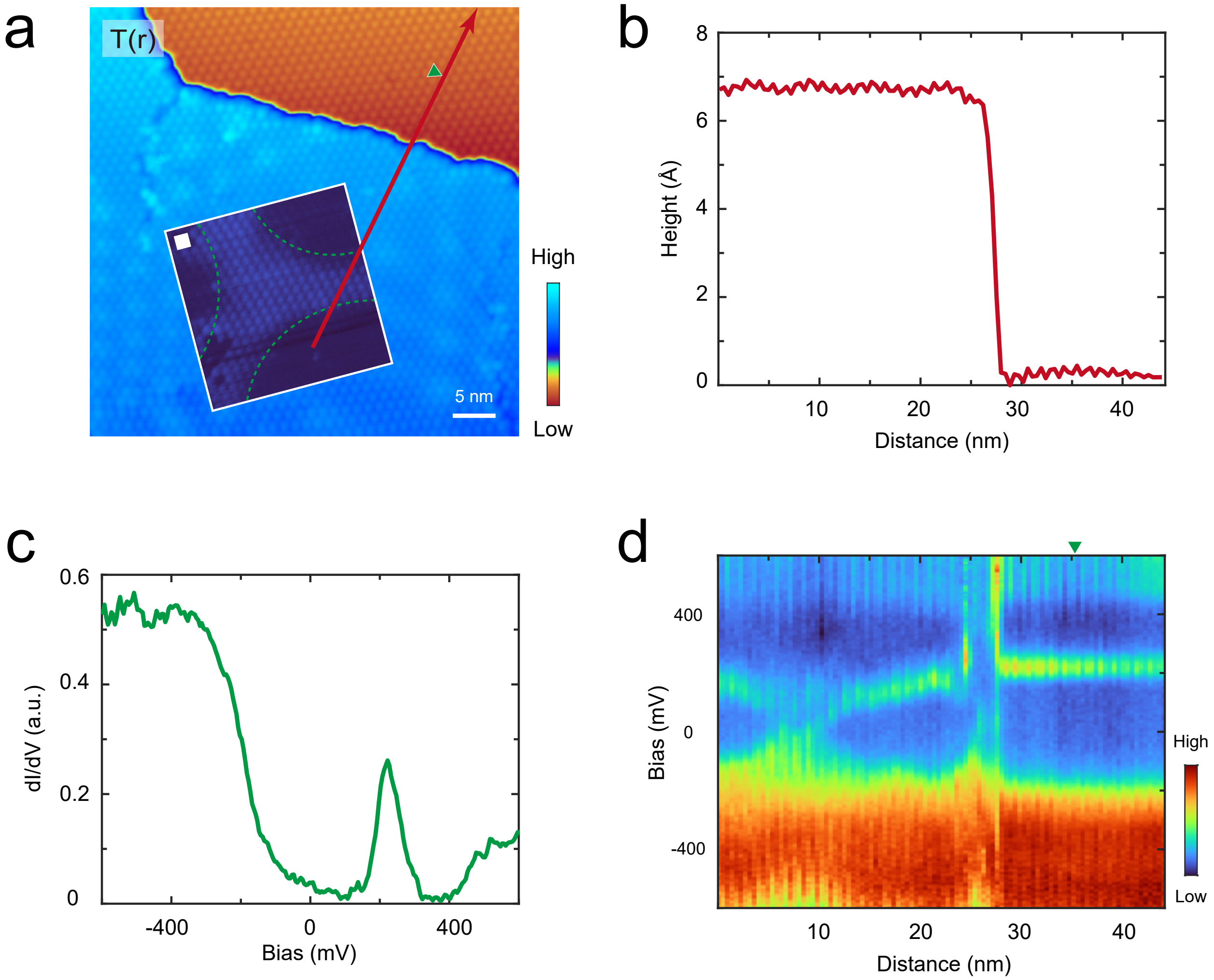

Fig. S3. **a**, The same topography as in Fig. S2. The white square frame indicates the zero-bias map shown in Fig. 3*D*. **b**, Height profile along the red arrowed line in **a**. **c**, A representative $\mathrm{d}I/\mathrm{d}V$ spectrum acquired at the green triangular marker along the red arrowed line and below the step. **d**, Distance-dependent $\mathrm{d}I/\mathrm{d}V$ spectra acquired along the red arrow in **a**.

Section S4

Horizontal displacement of the upper and lower SD centers along the moiré CDW superlattice direction

In this section, we investigate the horizontal displacement between the centers of SDs in the upper and lower layers along the directions of the SD moiré lattice, based on simulations with twist angles of 2.0° and 3.9°. Figure S4 **c** and **d** illustrate the evolution of this displacement along the yellow arrows indicated in Fig. S4 **a** and **b**, respectively. In both cases, similar trends are observed: the horizontal displacement of the upper and lower SD centers increases from zero, corresponding to ideal AA-type stacking, reaches a maximum value and then decreases back to nearly zero. The horizontal displacement for the AC-stacking is close to the maximum value in the SD moiré lattice.

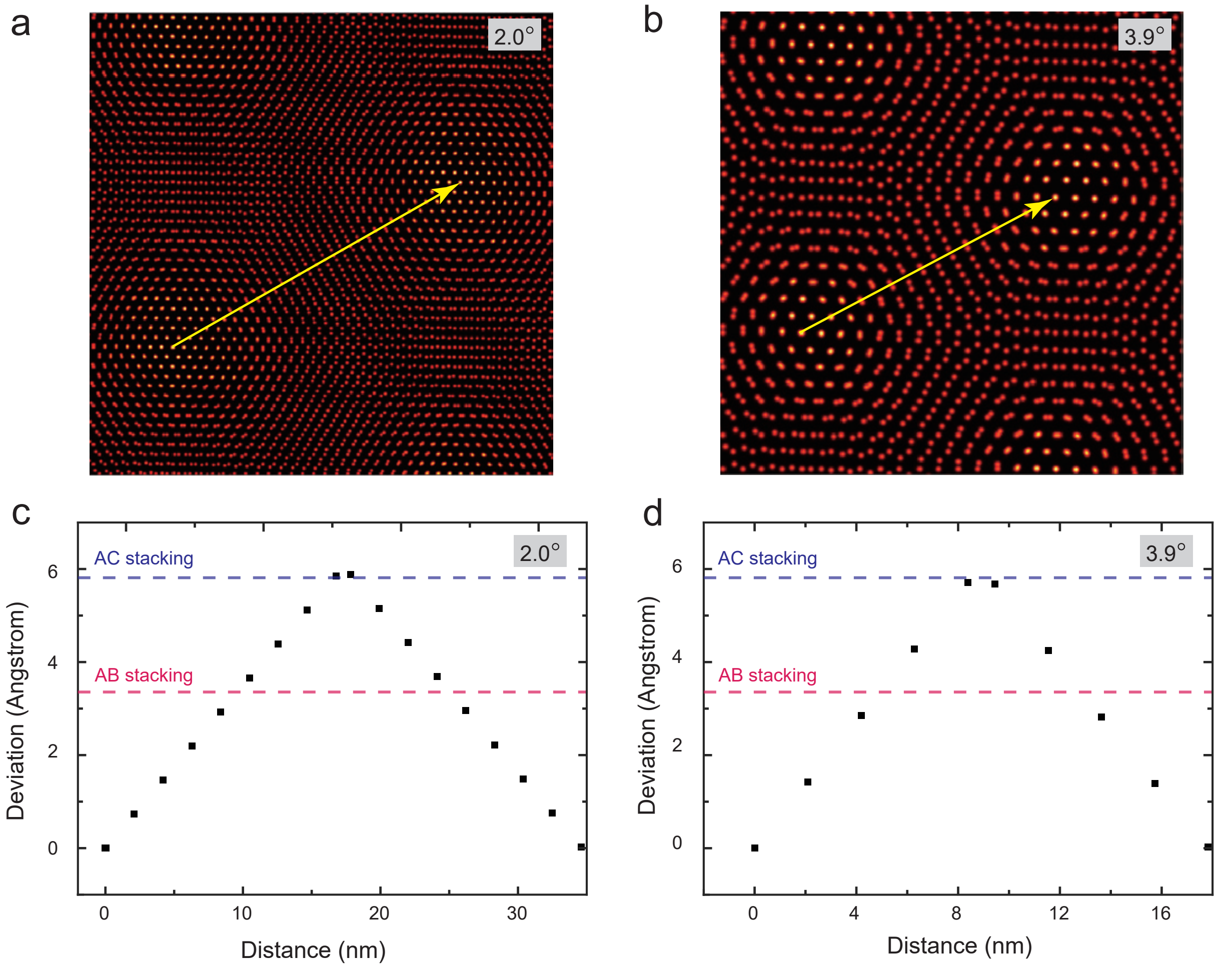

Fig. S4. **a**,**b** Simulated SD superlattices with twist angles of 2.0° and 3.9°, respectively. **c**,**d** Horizontal displacement between the upper and lower SD centers along the moiré CDW superlattice direction (yellow arrows) for the corresponding twist angles in **a** and **b**. The blue and red dashed lines mark the characteristic displacements for AC- and AB-type stacking configurations, respectively.

Section S5

DFT calculations of monolayer and bilayer 1T-$TaSe_2$

To further elucidate the role of strong electronic correlations in 1T-$TaSe_2$, we present comprehensive DFT results for monolayer and bilayer 1T-$TaSe_2$, as shown in Figs. S5 and S6, respectively. All calculations were performed along the high-symmetry momentum-space path $\Gamma - M - K - \Gamma$ within the Brillouin zone of the CDW superlattice. A plane wave cutoff energy of 400 eV was employed, with convergence thresholds for force and energy set to 0.01 eV/Å and 10^{-5} eV, respectively. Vacuum layers of approximately 15 Å were introduced to prevent spurious interactions between adjacent periodic images. The relaxed structure of 1T-$TaSe_2$ within the supercell yielded a lattice constant of 12.60 Å. A $4 \times 4 \times 1$ k-point grid was utilized for structural optimization and electronic structure calculations.

Figure S5 presents the band structures calculated within the DFT+U framework [1], where **a** and **b** correspond to the non-spinpolarized cases with U = 0 eV and U = 2 eV, respectively, and **c** and **d** correspond to the spin-polarized cases with U = 0 eV and U = 2 eV, respectively. We observe that, in the absence of spin polarization, the DFT+U calculation with U = 0 eV yields a relatively flat band near the Fermi level, originating from the CDW supercell. Even when increasing the Hubbard U parameter to U = 2 eV without including spin polarization, the resulting band structure remains largely unchanged compared to the U = 0 eV case, still exhibiting a flat band near the Fermi level. When spin polarization is included, the DFT results exhibit an energy gap opening near the Fermi level. Notably, even at U = 0 eV, a finite gap emerges around the Fermi level, although its magnitude is smaller compared to the case with U = 2 eV.

Spin polarization plays a crucial role in the opening of the energy gap. The exchange interaction induced by the Coulomb potential gives rise to distinct effective potential fields for spin-up and spin-down electrons, leading to energy-level splitting and energetic separation. According to the Stoner criterion, the flat band near the Fermi level undergoes spin splitting to minimize the systems total energy, with the spin-up band shifting downward in energy and the spin-down band shifting upward. At U = 0 eV, the spin-down band remains nearly dispersionless due to a large energy separation from higher-lying bands (above 0.4 eV) ,

whereas the spin-up band exhibits slightly greater dispersion as it lies close to lower-energy bands (below -0.1 eV). When electronic correlations are enhanced to U = 2 eV, the bands near the Fermi level experience further splitting; notably, the spin-up band below the Fermi level shifts downward and mixes with the lower-energy bands, resulting in only the flat bands above the Fermi level remaining clearly discernible. Spin-polarized DFT calculations for U = 2 eV yield a relatively large band gap in the monolayer system, consistent with the characteristic band gap structure of a Mott insulator. The spin-up and spin-down bands located below and above the Fermi level, respectively, correspond to the lower and upper Hubbard bands in the Mott insulating state.

The exchange interaction and strong electron correlation are fundamentally intertwined. Within the framework of DFT, the exchange-correlation interaction is approximately described by generalized gradient approximation (GGA) functionals, with the PBE functional we used as a representative example [2, 3, 4, 5], which accounts for the ability of spin-polarized DFT calculations to predict magnetic behavior. However, in systems where the Coulomb repulsion between electrons significantly exceeds their kinetic energy—characteristic of strongly correlated materials—standard GGA functionals tend to underestimate the strength of correlation effects. To address this limitation, the DFT+U method introduces an explicit local Coulomb repulsion parameter U into the DFT formalism, thereby improving the description of strongly correlated electronic states. In this study, the value of U = 2 eV is adopted empirically from the literature [6] and chosen to facilitate the analysis and interpretation of the phenomena under investigation.

For the bilayer coupled system, the calculated band structures corresponding to three distinct stacking configurations—AA, AB, and AC—are presented in the main text. In this DFT+U calculation, a Hubbard parameter of U = 2 eV was employed. Notably, we find that including or omitting spin polarization yields identical band structures for the bilayer system. This result arises from the antiferromagnetic alignment of spins in the upper and lower layers within the bilayer structure, which leads to a cancellation of net spin polarization. Nevertheless, the band gap still depends on the value of U. Figure S6 displays the computed band structures for all three stacking configurations at U = 0, 1, 2, and 4 eV. It is observed that while the overall band dispersion across different stackings remains largely unchanged,

the band gap increases monotonically with increasing U, indicating that intra-layer electron correlations play a subtle but existing role in modulating the overall electronic energy levels. The band tunability with respect to U demonstrates that the bilayer system cannot be described as a simple, trivial single-particle band system. However, when focusing on the key factor of hybridization-induced split flat bands, the band gap can still be extracted as a quantitative measure of the local hybridization or interlayer interaction strength. In the following section, we discuss the extraction of the spatially resolved gap map and its application as the interlayer interaction potential $V(\mathbf{r})$ in the constructing and interpretation of coupled moiré bands.

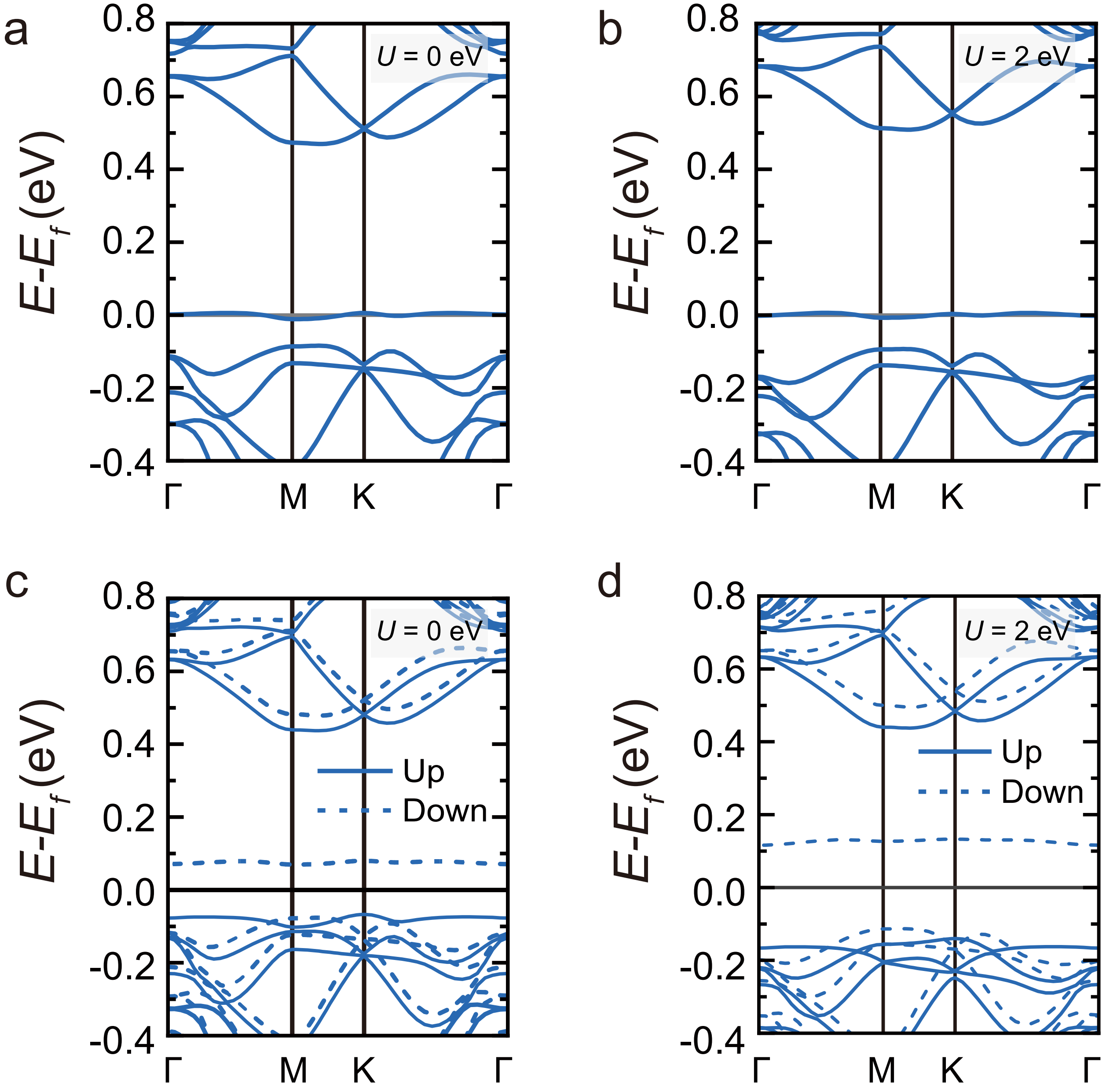

Fig. S5. **a**,**b** Non-spin-polarized band structure of monolayer 1T-$TaSe_2$ from DFT+U calculations with Hubbard U values of 0 eV (total energy = -276.176 eV) and 2 eV (total energy = -253.576 eV). **c**,**d** Band structure of monolayer 1T-$TaSe_2$ from spin-polarized DFT+U calculations with Hubbard U values of 0 eV (total energy = -276.189 eV) and 2 eV (total energy = -253.628 eV). Solid and dashed lines denote spin-up and spin-down channels, respectively. The minimum bandgap increases from 0.137 eV to 0.229 eV with the inclusion of the Hubbard U correction.

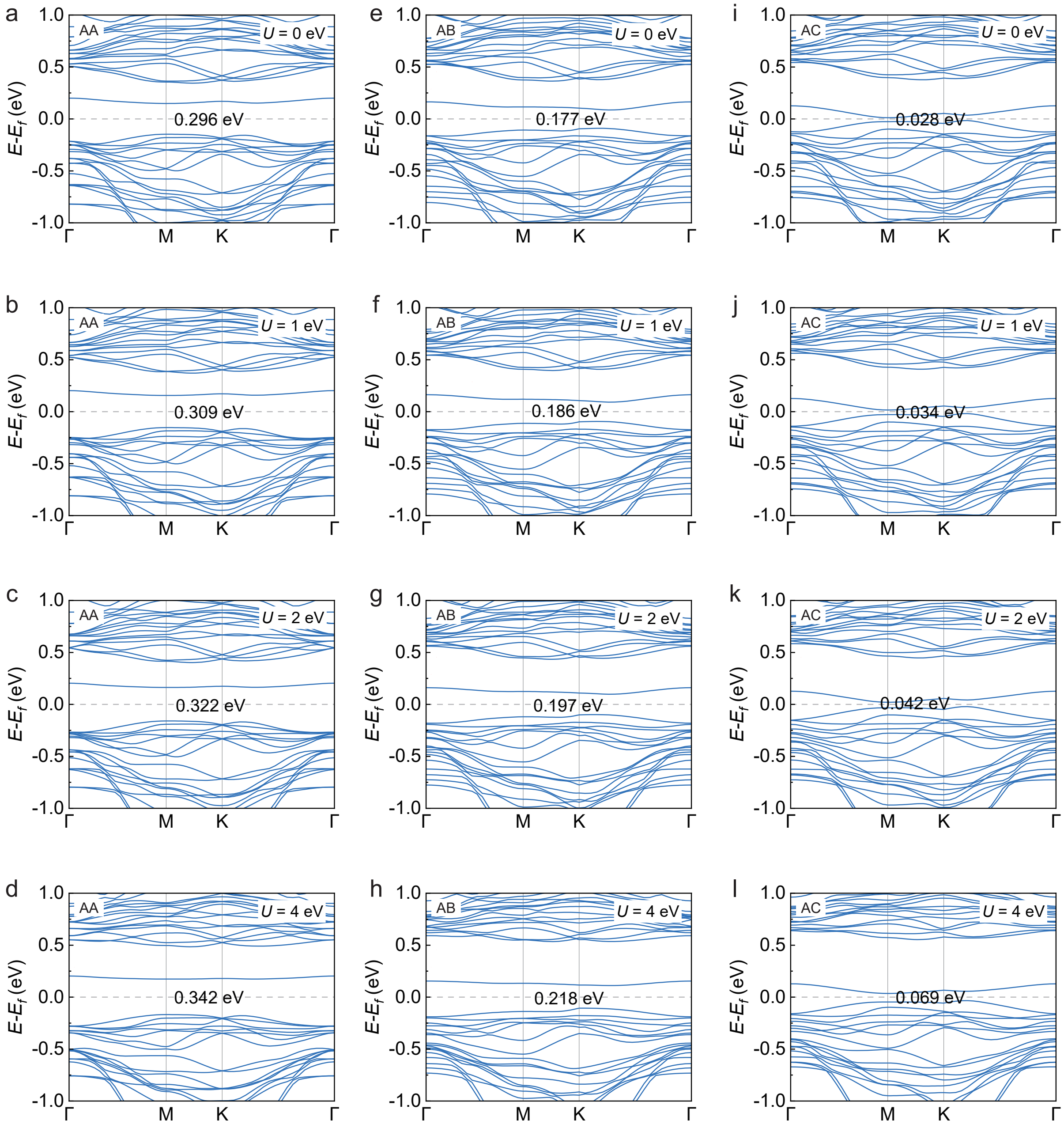

Fig. S6. Band structures of the **a-d**, AA-, **e-h**, AB-, and **i-l**, AC-stacking configurations calculated with Hubbard U values of 0, 1, 2, and 4 eV. The minimum energy separation for each case is explicitly indicated on the corresponding panel.

Section S6

Continuum model for the moiré bands of twisted bilayer $1T$-$TaSe_2$

Given the flat-band nature of monolayer $1T$-$TaSe_2$ near the Fermi level (in the absence of spin polarization), the bilayer system exhibits hybridization-induced split flat bands. The experimentally measured local spectral gap can thus serve as an indicator of interlayer coupling strength. For each spatial point in the scanned region (220×220 pixels, Fig. 3C of the main text), we extracted the positive peak position in the $\mathrm{d}I/\mathrm{d}V$ spectrum as the gap magnitude, generating a spatially resolved gap map of size 22 nm $\times$ 22 nm (Fig. S7 **a**).

In this gap map, a threefold-symmetric Y-shaped region appears at the center, corresponding to a metallic state with an artificially assigned zero gap. The boundary of the gapped region in the supper left corner shows a highly irregular morphology, likely due to impurity signals near the edge. The gapped area in the upper right corner is relatively small, while that in the lower right is substantially larger. Linecut data from the main text (Fig. 3E) further confirm that the gap increases from the edge toward the interior of the gapped region, exhibiting a saturation behavior.

Owing to the limited spatial extent of the original gap map, we performed a symmetry-based extrapolation to better visualize its distribution over a larger area. Using the center of the Y-shaped region as a reference point, the original map was rotated by integer multiples of 60° to generate six rotated sub-images. Each was translated by a distinct vector derived from the SD moiré pattern corresponding to a 2.0° interlayer twist. In overlapping regions, data were replaced by the average of the contributing sub-images. Figure S7 **b** shows the combined gap maps overlaid on the moiré pattern. The rotated square frames introduces a spurious chirality-like pattern in the image overlay, which is purely an artifact of the combined image. It should be emphasized that there is no intrinsic chirality or chiral feature present in our experimental results and theoretical interpretation.

Notably, although the gap in the lower-right region of Fig. S7 **a** reaches a large value, its location remains offset from the central AA-stacking region in the moiré pattern. In the central region, where experimental data are absent, the interlayer SD separation is minimal—comparable to ideal AA stacking—consistent with the saturation of the gap at the edge of

the scanned area. Furthermore, the multiple Y-shaped metallic regions in the combined gap maps correspond one-to-one with the positions of maximum interlayer separation in the SD moiré pattern.

The extracted gap map represents the spatially varying interlayer interaction potential $V(\mathbf{r})$, which retains the periodicity of the SD moiré pattern. We filled the missing central data in the combined gap map with a constant gap value of 240 meV (slightly larger than the 220 meV) and constructed a synthetic periodic function via symmetry-based replication and periodic extension. Figure S7 **c** shows a 120 nm $\times$ 120 nm region of this periodic $V(\mathbf{r})$. The Fourier transform of a 1000 nm $\times$ 1000 nm section, displayed in Fig. S7 **d**, yields $V(\mathbf{q})$. Owing to the periodicity of $V(\mathbf{r})$, its Fourier transform $V(\mathbf{q})$ is discrete, taking nonzero values only at the reciprocal lattice vectors $\mathbf{G}$ of the SD moiré superlattice.

All reciprocal lattice vectors can be expressed as $\mathbf{G} = m\mathbf{b}_{\mathrm{M}}^1 + n\mathbf{b}_{\mathrm{M}}^2$, where m and n are integers, and $\mathbf{b}_{\mathrm{M}}^1$ and $\mathbf{b}_{\mathrm{M}}^2$ are the moiré reciprocal basis vectors with a 120° angle between them. In Fig. S7(d), the origin ($\mathbf{G}_0 = 0$, red circle) gives $V_{\mathbf{G}_0} = 0.1564$ eV. The first-order peaks (yellow circles) correspond to the nearest-neighbor reciprocal lattice points, forming a regular hexagon defined by the three basis vectors $\mathbf{G}_1 = \mathbf{b}_{\mathrm{M}}^1$, $\mathbf{G}_2 = \mathbf{b}_{\mathrm{M}}^2$, $\mathbf{G}_3 = -\mathbf{b}_{\mathrm{M}}^1 - \mathbf{b}_{\mathrm{M}}^2$, and their opposites. The corresponding Fourier components are $V_{\pm\mathbf{G}_1} = V_{\pm\mathbf{G}_2} = V_{\pm\mathbf{G}_3} = 0.0259$ eV. The second-order peaks (green circles) yield values of 0.0125 eV. For twisted $1T$-$TaSe_2$, we focus on using the positive peak to extract $\Delta(\mathbf{r})$, due to the well-isolated and clean spectral feature of the upper flat band. The lower flat band is mixed with other dispersive bands, leading to an inaccurate estimation of the flat band position from the negative peak in the $\mathrm{d}I/\mathrm{d}V$ spectra. When the gap map is extracted from the negative peak position, the corresponding calculated $V_{\mathbf{G}_0}$ is found to be larger, leading to increased band splitting, as expected.

We construct a low-energy continuum model for the triangular SD superlattice in $1T$-$TaSe_2$, following previous approaches [7, 8, 9]. For simplicity, we consider only interlayer scattering mediated by the leading Fourier components of the interaction potential: $V_{\mathbf{G}_0}$,$V_{\mathbf{G}_1}$, $V_{\mathbf{G}_2}$,and $V_{\mathbf{G}_3}$. The Hamiltonian is given by $H = h_1 + h_2 + H_{\mathrm{in}}$, where h_1 and h_2 describe the isolated monolayers, and H_{in} represents the interlayer interaction (or tunneling), expressed in terms of the Fourier components $V_{\mathbf{G}}$ introduced above.

The monolayer band structure of $1T$-$TaSe_2$ is obtained from spin-unpolarized DFT+U calculations with U = 0 eV, focusing on the flat band near the Fermi level. Due to computational constraints, the DFT calculations were performed along one-dimensional paths connecting high-symmetry points in the Brillouin zone. To construct the continuum model, we fit these results using a four-parameter tight-binding model, which allows us to interpolate the band dispersion across the full two-dimensional Brillouin zone and build an effective single-band Hamiltonian. The tight-binding model is described by,

$$h = \varepsilon_0 - \sum_{<i,j>} t_N c_i^\dagger c_j - \sum_{<<i,j>>} t_{NN} c_i^\dagger c_j - \sum_{<<<i,j>>>} t_{NNN} c_i^\dagger c_j + h.c., \tag{3}$$

where $c_i^\dagger$ (c_i) creates (annihilates) an electron on the $5d$ orbital of the ith SD, ε_0 denotes the on-site energy, and t_N, t_{NN} and t_{NNN} represent the nearest-neighbor, next-nearest-neighbor and next-next-nearest-neighbor hopping amplitudes, respectively. Fitting the DFT-derived flat band with the tight-binding model yields the following parameters: $\varepsilon_0 = 0.612$ meV, $t_N = 1.179$ meV, $t_{NN} = 0.214$ meV, and $t_{NNN} = -1.461$ meV. The flat-band character is reflected in the relatively large magnitude of the next-next-nearest-neighbor hopping t_{NNN} compared to t_N and t_{NN}. Using this parameterized model, we can interpolate the band energy at arbitrary wavevectors across the two-dimensional Brillouin zone.

According to the generalized umklapp condition [10], two states with momenta $\mathbf{k}_1$ and $\mathbf{k}_2$ in layers 1 (bottom) and 2 (upper), respectively, are coupled only if their exist reciprocal lattice vectors $\mathbf{B}_1 = m_1\mathbf{b}_1^1 + n_1\mathbf{b}_1^2$ and $\mathbf{B}_2 = m_2\mathbf{b}_2^1 + n_2\mathbf{b}_2^2$ such that $\mathbf{k}_1 + \mathbf{B}_1 = \mathbf{k}_2 + \mathbf{B}_2$, where $\mathbf{b}_j^i$ denotes the reciprocal basis vectors of layer j. The momentum difference can generally be written as $\mathbf{k}_2 - \mathbf{k}_1 = m(\mathbf{b}_2^1 - \mathbf{b}_1^1) + n(\mathbf{b}_2^2 - \mathbf{b}_1^2)$. Given the rapid decay of $V_\perp(\mathbf{k})$ with increasing $|\mathbf{k}|$ [7, 11] and the small twist angle, we impose the constraints $m_1 = m_2 = -m$ and $n_1 = n_2 = -n$, leading to $\mathbf{k}_2 - \mathbf{k}_1 = m(\mathbf{b}_2^1 - \mathbf{b}_1^1) + n(\mathbf{b}_2^2 - \mathbf{b}_1^2)$. Defining the morié reciprocal basis vectors as $\mathbf{b}_\mathrm{M}^1 = \mathbf{b}_2^1 - \mathbf{b}_1^1$ and $\mathbf{b}_\mathrm{M}^2 = \mathbf{b}_2^2 - \mathbf{b}_1^2$, we obtain $\mathbf{k}_2 - \mathbf{k}_1 = m\mathbf{b}_\mathrm{M}^1 + n\mathbf{b}_\mathrm{M}^2$. We retain only the four dominant interlayer scattering processes corresponding to $\mathbf{k}_2 - \mathbf{k}_1 = \mathbf{G}_0, \mathbf{G}_1, \mathbf{G}_2, \mathbf{G}_3$, where $\mathbf{G}_0 = 0$, $\mathbf{G}_1 = \mathbf{b}_\mathrm{M}^1$, $\mathbf{G}_2 = \mathbf{b}_\mathrm{M}^2$, and $\mathbf{G}_3 = -\mathbf{b}_\mathrm{M}^1 - \mathbf{b}_\mathrm{M}^2$, as illustrated in Fig. S8 **a**. This approximation is equivalent to including only the Fourier components $V_{\mathbf{G}_0}$, $V_{\mathbf{G}_1}$, $V_{\mathbf{G}_2}$, and

$V_{\mathbf{G}_3}$ in the bilayer interaction. The strength of these interaction terms are obtained by expanding $V(\mathbf{r})$ into its Fourier components up to the first harmonic:

$$V(\mathbf{r}) = V_{\mathbf{G}_0} + \sum_{i=1}^{3} V_{\mathbf{G}_i} e^{i\mathbf{G}_i \cdot \mathbf{r}}. \tag{4}$$

For simplicity, contributions from $V_{-\mathbf{G}_1}$, $V_{-\mathbf{G}_2}$, and $V_{-\mathbf{G}_3}$ are neglected, but this still allows us to capture the essential features of the moiré bands in the following two examples.

We first consider an electronic state $\mathbf{k}$ in layer 1 that is scattered into four distinct states in layer 2. The corresponding Hamiltonian matrix is constructed as follows:

$$H(\mathbf{k}_1, \theta) = \begin{bmatrix} h_1(\mathbf{k}_1) & V_{\mathbf{G}_0} & V_{\mathbf{G}_1} & V_{\mathbf{G}_2} & V_{\mathbf{G}_3} \\ V_{\mathbf{G}_0} & h_2^{\theta}(\mathbf{k}_1 + \mathbf{G}_0) & 0 & 0 & 0 \\ V_{\mathbf{G}_1} & 0 & h_2^{\theta}(\mathbf{k}_1 + \mathbf{G}_1) & 0 & 0 \\ V_{\mathbf{G}_2} & 0 & 0 & h_2^{\theta}(\mathbf{k}_1 + \mathbf{G}_2) & 0 \\ V_{\mathbf{G}_3} & 0 & 0 & 0 & h_2^{\theta}(\mathbf{k}_1 + \mathbf{G}_3) \end{bmatrix}$$

where θ is the twist angle between the two layers. The Hamiltonian acts on a five-component state vector $\Psi = (\phi_1, \phi_2^0, \phi_2^1, \phi_2^2, \phi_2^3)^{\mathrm{T}}$, where ϕ_1 corresponds to the electronic state in layer 1 and the remaining components represent the four scattered states in layer 2. Using the fitted tight-binding model, we compute the original flat band and the shifted bands in the twisted layer ($\theta = 2.0°$), as shown in Fig. S8 **b**. The y-axis is magnified to resolve the fine features of the flat band. Although the original flat band shows weak dispersion, the momentum-shifted bands exhibit slight energy splitting relative to the original band.

Diagonalizing the Hamiltonian for different values of $\mathbf{k}_1$ yields the band structure of twisted $1T$-$TaSe_2$, as shown in Fig. S8 **c**. The initial five flat bands near E_{F} are reconstructed into two split flat bands near ± 0.16 eV and three bands remaining at the Fermi level. The

wavefunctions for electronic states at the K point are given below:

$$\Psi_1^K = \begin{bmatrix} 0.71 \\ -0.68 \\ -0.11 \\ -0.11 \\ -0.11 \end{bmatrix} \quad \Psi_2^K = \begin{bmatrix} 0.71 \\ 0.68 \\ 0.11 \\ 0.11 \\ 0.11 \end{bmatrix} \quad \Psi_3^K = \begin{bmatrix} 0 \\ -0.16 \\ -0.03 \\ 0.99 \\ -0.02 \end{bmatrix} \quad \Psi_4^K = \begin{bmatrix} 0 \\ -0.16 \\ -0.02 \\ 0 \\ 0.99 \end{bmatrix} \quad \Psi_5^K = \begin{bmatrix} 0 \\ -0.16 \\ 0.99 \\ 0 \\ 0 \end{bmatrix}.$$

The two split flat bands near ± 0.16 eV originate primarily from the hybridization between the original band in layer 1 (black solid line in Fig. S8 **b**) and the rotated band in layer 2 (red dash-dotted line). The significant energy splitting is due to the relatively large zero-momentum scattering term $V_{\mathbf{G}_0}$, while the weaker terms $V_{\mathbf{G}_1}$, $V_{\mathbf{G}_2}$, and $V_{\mathbf{G}_3}$ leave the three remaining bands near E_{F} largely unchanged. These three bands do not hybridize with each other, and thus exhibit no splitting. Their superposition states show only weak mixing with other states, mediated by the $V_{\mathbf{G}_{1,2,3}}$ coupling to the original band in layer 1.

In the second example, we consider the electron states with momenta $\mathbf{k}_1 + \mathbf{G}_1$, $\mathbf{k}_1 + \mathbf{G}_2$ and $\mathbf{k}_1 + \mathbf{G}_3$ in layer 2 (rotated frame), which are scattered back to the corresponding states with the same momenta in layer 1 (unrotated frame) through the interaction term $V_{\mathbf{G}_0}$. An 8×8 Hamiltonian is constructed as follows:

$$H(\mathbf{k}_1, \theta) = \begin{bmatrix} h_1 & V_{\mathbf{G}_0} & V_{\mathbf{G}_1} & V_{\mathbf{G}_2} & V_{\mathbf{G}_3} & 0 & 0 & 0 \\ V_{\mathbf{G}_0} & h_2^\theta & 0 & 0 & 0 & 0 & 0 & 0 \\ V_{\mathbf{G}_1} & 0 & h_2^\theta & 0 & 0 & V_{\mathbf{G}_0} & 0 & 0 \\ V_{\mathbf{G}_2} & 0 & 0 & h_2^\theta & 0 & 0 & V_{\mathbf{G}_0} & 0 \\ V_{\mathbf{G}_3} & 0 & 0 & 0 & h_2^\theta & 0 & 0 & V_{\mathbf{G}_0} \\ 0 & 0 & V_{\mathbf{G}_0} & 0 & 0 & h_1 & 0 & 0 \\ 0 & 0 & 0 & V_{\mathbf{G}_0} & 0 & 0 & h_1 & 0 \\ 0 & 0 & 0 & 0 & V_{\mathbf{G}_0} & 0 & 0 & h_1 \end{bmatrix},$$

in which h_1 and h_2^θ are written in a simplified form. The bands structure obtained from diagonalization is shown in Fig. S8 **c**. The inclusion of additional $V_{\mathbf{G}_0}$ coupling clearly leads to further splitting of the flat bands. The following table lists the eigenenergies and

eigenstates for the above Hamiltonian.

Table S1: Eigenenergies E_i and eigenstates Ψ_i^K for the 8×8 Hamiltonian.

E_i **(eV)**	$\Psi_i^K = (v_1, v_2, \ldots, v_8)^{\mathrm{T}}$
$E_1 = 0.182$	$(0.533, 0.461, 0.306, 0.323, 0.299, 0.265, 0.282, 0.258)^{\mathrm{T}}$
$E_2 = 0.158$	$(0.023, -0.023, -0.363, 0.569, -0.209, -0.361, 0.572, -0.207)^{\mathrm{T}}$
$E_3 = 0.158$	$(0.012, 0.012, -0.450, -0.089, 0.538, -0.451, -0.090, 0.537)^{\mathrm{T}}$
$E_4 = 0.137$	$(0.464, 0.535, -0.269, -0.252, -0.278, -0.311, -0.293, -0.319)^{\mathrm{T}}$
$E_5 = -0.134$	$(-0.461, 0.533, -0.264, -0.284, -0.256, 0.305, 0.325, 0.297)^{\mathrm{T}}$
$E_6 = -0.154$	$(-0.023, 0.023, -0.363, 0.570, -0.208, 0.365, -0.568, 0.210)^{\mathrm{T}}$
$E_7 = -0.155$	$(-0.012, 0.012, 0.449, 0.090, -0.539, -0.448, -0.089, 0.540)^{\mathrm{T}}$
$E_8 = -0.179$	$(0.536, -0.464, -0.310, -0.295, -0.318, 0.269, 0.254, 0.277)^{\mathrm{T}}$

The eigenstate results confirms that the split flat bands at different eigenenergies correspond to distinct superpositions of multiple $\mathbf{k}+\mathbf{G}$ states from both layers. Although the $V_{\mathbf{G}_1}$, $V_{\mathbf{G}_2}$, and $V_{\mathbf{G}_3}$ terms are relatively weak, they play a crucial role in incorporating multiple momentum-transferred states, such as $\mathbf{k}+\mathbf{G}$, $\mathbf{k}+\mathbf{G}+\mathbf{G}'$, and higher-order combinations, into successive scattering processes. These results indicate that the moiré band structure of the twisted bilayer flat-band system comprises a large number of split flat bands in momentum space.

In bilayer graphene (with Dirac bands) or TMDs (with parabolic bands), multi-step interlayer scattering mediated by momentum transfer $\mathbf{G}$ decays rapidly due to increasing energy separation as momentum deviates from the initial point. In contrast, the monolayer flat band in $1T$-$TaSe_2$—arising from the CDW supercell—maintains near energy degeneracy even under successive $\mathbf{G}$-shifts, enabling iterative multiple scattering. As a result, the eigenstates are superpositions of distinct momentum-transfered states, producing multiple split flat bands with different energy gaps. This momentum-space superpositions is consistent with the formation of localized gapped states in real space, as expected from the Heisenberg uncertainty principle.

Figure S8 **b** also shows that slight energy differences between momentum-shifted states can lead to energy crossings, which may in turn produce even flatter bands, even after the original band splits into two separated subbands. This behavior is consistent with the formation of a superflat band when two flat bands are twisted relative to each other. The

nature of such superflat bands warrants further experimental investigation under ultralow-temperature conditions.

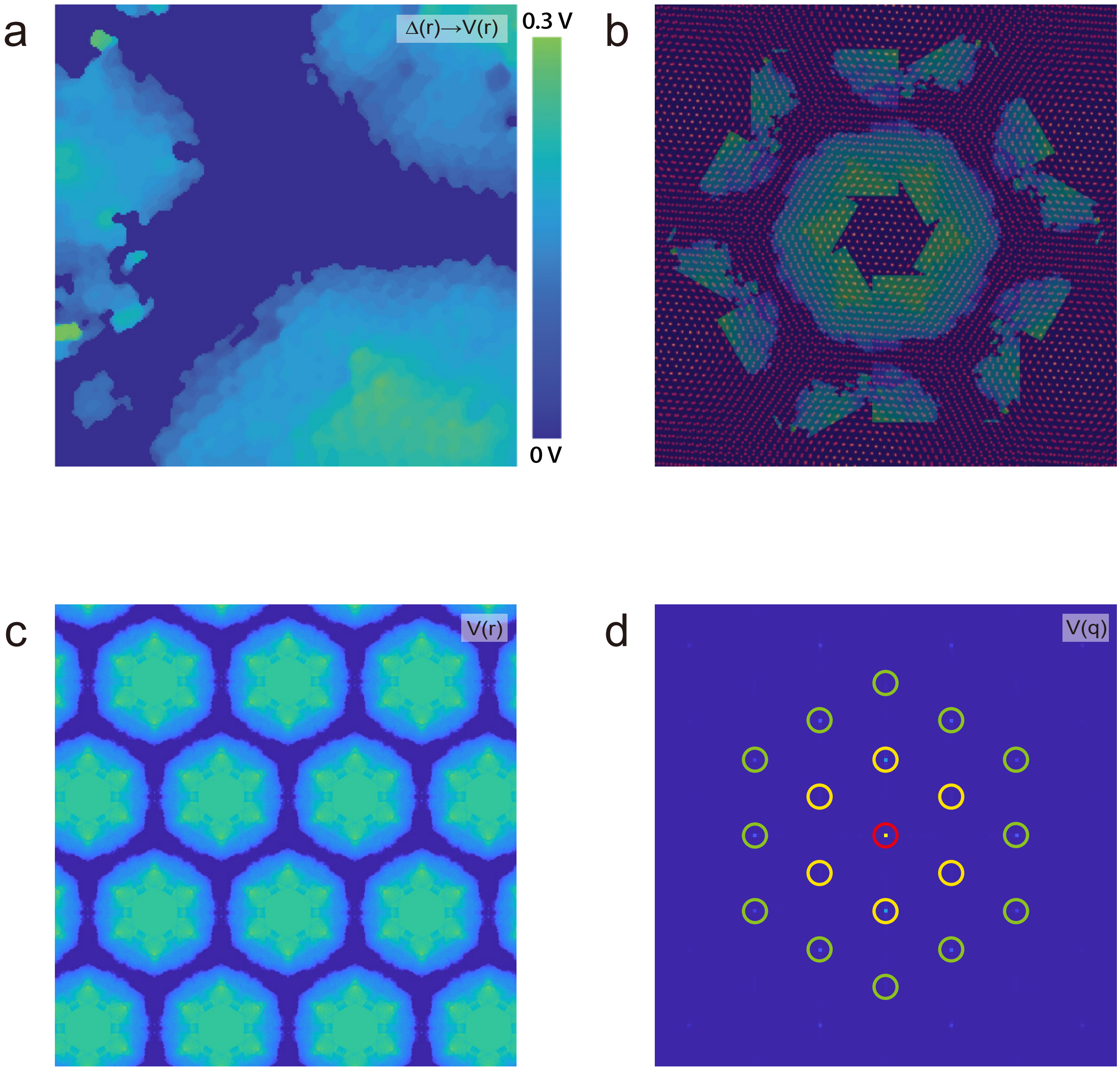

Fig. S7. **a**, Gap map of the twisted bilayer region in Fig. 3C with a twist angle of $\theta = 2°$ and a spatial extent of 22 nm × 22 nm. The gap value is determined from the positive peak position for gapped spectra, while metallic regions are assigned a value of zero. **b**, Six rotated gap maps overlaid on the simulated moiré pattern of bilayer SD superlattices. **c**, Symmetrically extended periodic gap map (120 nm × 120 nm) constructed from **a** and **b**. **d**, Fourier transform $V(\mathbf{q})$ of a 1000 nm × 1000 nm gap map. The origin (red circle), inner hexagonal peaks (yellow), and outer peaks (green) are highlighted.

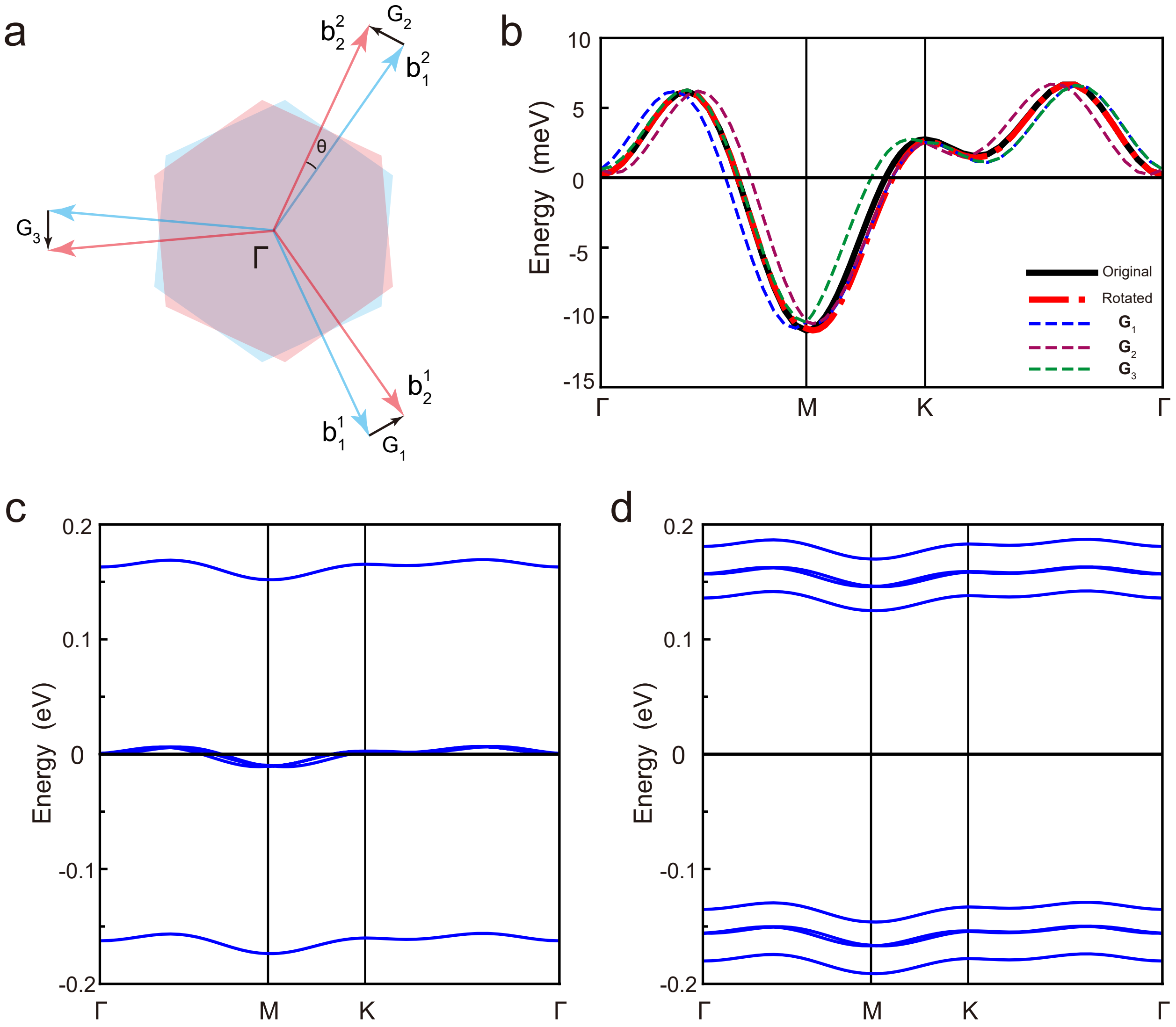

Fig. S8. **a**, Schematic of the twisted Brillouin zones for the lower (blue) and upper (red) layers, showing reciprocal basis vectors $\mathbf{b}^i_{1,2}$ and moiré reciprocal basis vectors $\mathbf{G}_{1,2,3}$. **b**, Magnified view of flat bands near E_F (the Fermi energy). The black solid and the red dash-dot lines are the original flat bands of layer 1 and 2 ($\theta = 2°$), respectively. Blue, purple, and green dashed lines indicates electronic momentum shifts by $\mathbf{G}_1$, $\mathbf{G}_2$, and $\mathbf{G}_3$ relative to $\mathbf{k}$. **c**,**d** Band structure from the 5×5 and 8×8 continuum models for twisted bilayer $1T$-$\mathrm{TaSe_2}$ with a twist angle of $\theta = 2°$, respectively.

References

[1] S. L. Dudarev, G. A. Botton, S. Y. Savrasov, C. J. Humphreys, A. P. Sutton, Electron-energy-loss spectra and the structural stability of nickel oxide: An LSDA+U study. *Phys. Rev. B* **57**, 1505 (1998).

[2] G. Kresse, J. Hafner, Ab initio molecular dynamics for liquid metals. *Phys. Rev. B* **47**, 558 (1993).

[3] G. Kresse, J. Furthmller, Efficient iterative schemes for ab initio total-energy calculations using a plane-wave basis set. *Phys. Rev. B* **54**, 11169 (1996).

[4] J. P. Perdew, J. A. Chevary, S. H. Vosko, K. A. Jackson, M. R. Pederson, D. J. Singh, Atoms, molecules, solids, and surfaces: Applications of the generalized gradient approximation for exchange and correlation. *Phys. Rev. B* **46**, 6671 (1996).

[5] P. E. Blöchl, Projector augmented-wave method. *Phys. Rev. B* **50**, 17953 (1994).

[6] Y. Chen *et al.*, Strong correlations and orbital texture in single-layer $1T$-$TaSe_2$. *Nat. Phys.* **16**, 218-224 (2020).

[7] R. Bistritzer, A. H. MacDonald, Moiré bands in twisted double-layer graphene. *Proc. Natl. Acad. Sci. U.S.A.* **108**, 12233-12237(2011).

[8] F. Wu, T. Lovorn, E. Tutuc, I. Martin, A. H. MacDonald, Topological insulators in twisted transition metal dichalcogenide homobilayers. *Phys. Rev. Lett.* **122**, 086402 (2019).

[9] Y. Zhang, T. Liu, L. Fu, Electronic structures, charge transfer, and charge order in twisted transition metal dichalcogenide bilayers. *Phys. Rev. B* **103**, 155142 (2021).

[10] M. Koshino, Interlayer interaction in general incommensurate atomic layers. *New J. Phys.* **17**, 015014 (2015).

[11] V. M. Pereira, A. H. Castro Neto, N. M. R. Peres, Tight-binding approach to uniaxial strain in graphene. *Phys. Rev. B* **80**, 045401 (2009).
